# Influenza surveillance in Europe: comparing intensity levels calculated using the moving epidemic method

**DOI:** 10.1111/irv.12330

**Published:** 2015-08-04

**Authors:** Tomás Vega, José E Lozano, Tamara Meerhoff, René Snacken, Julien Beauté, Pernille Jorgensen, Raúl Ortiz de Lejarazu, Lisa Domegan, Joël Mossong, Jens Nielsen, Rita Born, Amparo Larrauri, Caroline Brown

**Affiliations:** a Public Health Directorate, Castilla y León Regional Health MinistryValladolid, Spain; b The Radboud University Nijmegen Medical CentreNijmegen, The Netherlands; c European Centre for Disease Prevention and ControlStockholm, Sweden; d Division of Health Security, Infectious Diseases and the Environment, WHO Regional Office for EuropeCopenhagen, Denmark; e National Influenza Centre, University of ValladolidValladolid, Spain; f Health Protection Surveillance CentreDublin, Ireland; g Laboratoire National de SantéLuxembourg, Luxembourg; h Statens Serum InstituteCopenhagen, Denmark; i Division of Communicable Diseases, Federal Office of Public Health, Directorates of Public HealthBern, Switzerland; j CIBER Epidemiología y Salud Pública (CIBERESP), Institute of Health Carlos IIIMadrid, Spain

**Keywords:** Incidence, influenza-like illnesses, primary care, surveillance

## Abstract

**Objectives:**

Although influenza-like illnesses (ILI) and acute respiratory illnesses (ARI) surveillance are well established in Europe, the comparability of intensity among countries and seasons remains an unresolved challenge. The objective is to compare the intensity of ILI and ARI in some European countries.

**Design and setting:**

Weekly ILI and ARI incidence rates and proportion of primary care consultations were modeled in 28 countries for the 1996/1997–2013/2014 seasons using the moving epidemic method (MEM). We calculated the epidemic threshold and three intensity thresholds, which delimit five intensity levels: baseline, low, medium, high, and very high. The intensity of 2013/2014 season is described and compared by country.

**Results:**

The lowest ILI epidemic thresholds appeared in Sweden and Estonia (below 10 cases per 100 000) and the highest in Belgium, Denmark, Hungary, Poland, Serbia, and Slovakia (above 100 per 100 000). The 2009/2010 season was the most intense, with 35% of the countries showing high or very high intensity levels. The European epidemic period in season 2013/2014 started in January 2014 in Spain, Poland, and Greece. The intensity was between low and medium and only Greece reached the high intensity level, in weeks 7 to 9/2014. Some countries remained at the baseline level throughout the entire surveillance period.

**Conclusions:**

Epidemic and intensity thresholds varied by country. Influenza-like illnesses and ARI levels normalized by MEM in 2013/2014 showed that the intensity of the season in Europe was between low and medium in most of the countries. Comparing intensity among seasons or countries is essential for understanding patterns in seasonal epidemics. An automated standardized model for comparison should be implemented at national and international levels.

## Introduction

Influenza and other respiratory virus infections are the most common causes of primary care consultation and represent an important economic burden worldwide.^1^–^4^ In a typical season, annual attack rate of influenza is estimated at 5–10% in adults and 20–30% in children,^5^ although not all cases seek medical care and are captured by the surveillance systems.

It is widely accepted that influenza surveillance should address the following objectives: monitoring the circulating virus strains, the timing, intensity and severity of the epidemic waves, providing information about the underlying risk conditions associated with severity as well as supplying epidemiological and virological support for pandemic early warning and preparedness.^6^

Influenza surveillance is supported by quantitative and qualitative indicators aimed at assessing the burden of seasonal epidemics. Such indicators are principally based on clinical consultations in general practice, hospitalized laboratory-confirmed cases, sentinel and non-sentinel positive specimens, mortality, and local outbreaks. One of the most important indicators in influenza surveillance is the estimated incidence or percentage of consultations in a population in a given period, which are related to the intensity of seasonal epidemics. Overall seasonal or weekly influenza intensity levels are useful for understanding the dynamics of influenza epidemics and for comparing trends within a country or intercountry differences. However, evaluating the impact of prevention and control measures^7^ needs a reliable morbidity assessment.

Influenza-like illness (ILI) and acute respiratory infection (ARI) population-based consultation rates in primary care settings are the most common quantitative indicators in Europe and other developed countries, in either sentinel or universal surveillance systems. The percentage of consultations due to ILI or ARI is used in countries where a population denominator is not available.

In the WHO European Region, intensity is reported, via web-based platforms, as a qualitative indicator based on the overall level of clinical ILI or ARI consultations in the country or at subnational level, in comparison with the historical data available.^8^ The ILI or ARI consultation rate or weekly percentage of consultations due to ILI or ARI are subjectively evaluated by the local epidemiologist as low (no activity or activity at the baseline level), medium (usual levels of activity), high (levels of activity higher than usual), and very high (exceptionally high levels of activity). In the United States of America, intensity levels are divided into four categories (minimal, low, moderate, and high) according to the number of standard deviations above the national baseline (average percent of ILI visits that occur during weeks with little or no influenza virus circulation).^9^

National and international institutions have implemented surveillance systems for which the comparability of indicators (rates, intensity, trend, geographic spread, etc.) is an important goal. Several methods to establish epidemic thresholds and intensity levels have been developed and are being used in an increasing number of countries.^10^–^13^ With their advantages and limitations, these methods could be used for regional, national, and international comparison, provided that they are calculated with the same mathematical methods and parameters over time. The moving epidemic method (MEM) model^14^ is being used in web platforms in Europe for weekly reports. This pilot experience is based on data collected through the European Influenza Surveillance Network (EISN), under coordination by the European Centre for Disease Prevention and Control (ECDC), Sweden, since 2011/2012 and through the EuroFlu platform (WHO Regional Office for Europe), Denmark, since 2012/2013. Primarily developed to detect the beginning of epidemic periods through ‘epidemic thresholds,’ MEM calculates different ‘intensity thresholds’ to weekly monitor the intensity level of the ILI or ARI waves. The experience accumulated in these three seasons has made it possible to review the methods, explore more output possibilities, and look at the benefit of having this tool at the national and international levels.

The objectives of this article were to describe the epidemic and intensity thresholds of ILI and ARI calculated by MEM and the intensity of influenza seasons in some European countries. We also discuss whether these indicators accurately summarize the season epidemics and the usefulness of this method for comparisons at the national and international levels.

## Methods

Weekly ILI and ARI incidence rates or proportions of primary care consultations from the 1996/1997 to the 2013/2014 influenza season (from week 40 to week 20 of the next year) were taken from the EuroFlu database (WHO Regional Office for Europe). Countries included in the study were selected among the 50 participating Member States in the WHO European Region, according to the following criteria: data available for at least six consecutive seasons, excluding the pandemic season 2009/2010 (a minimum of five seasons for the calculations and the target season), and no major changes in the surveillance systems during the reporting period. Countries fulfilling these criteria were invited to participate.

Data were checked for inconsistencies, such as abnormal weekly estimates or missing values during the surveillance period, and sent to the country representatives for validation and updating when necessary.

A sequential analysis using the R Language implementation of MEM (package “mem” [Internet]. Available from: http://cran.r-project.org/web/packages/mem/index.html) was carried out for each country to calculate the epidemic threshold (level of influenza activity that signals the start and end of the annual epidemic wave) and the three intensity thresholds (medium, high, and very high) for each season. The number of seasons included in each analysis ranged from five to 10 (training period).

Moving epidemic method has three main steps, which have been previously described.^14^ In the first step, for each season separately, the length of the epidemic period is estimated as the minimum number of consecutive weeks with the maximum accumulated percentage rates, splitting the season in three periods: a pre-epidemic, an epidemic, and a post-epidemic period. In the second step, MEM calculates the epidemic threshold as the upper limit of the 95% one-sided confidence interval of 30 highest pre-epidemic weekly rates, the *n* highest for each season taking the whole training period, was *n* = 30/number of seasons. In the third step, medium, high, and very high intensity thresholds were estimated as the upper limits of the 40%, 90%, and 97·5% one-sided confidence intervals of the geometric mean of 30 highest epidemic weekly rates, the *n* highest for each season taking the whole training period, were *n* = 30/number of seasons. For the purposes of this work, if the medium intensity threshold is lower than the epidemic threshold, the epidemic threshold is used for both.

**Figure 1:**
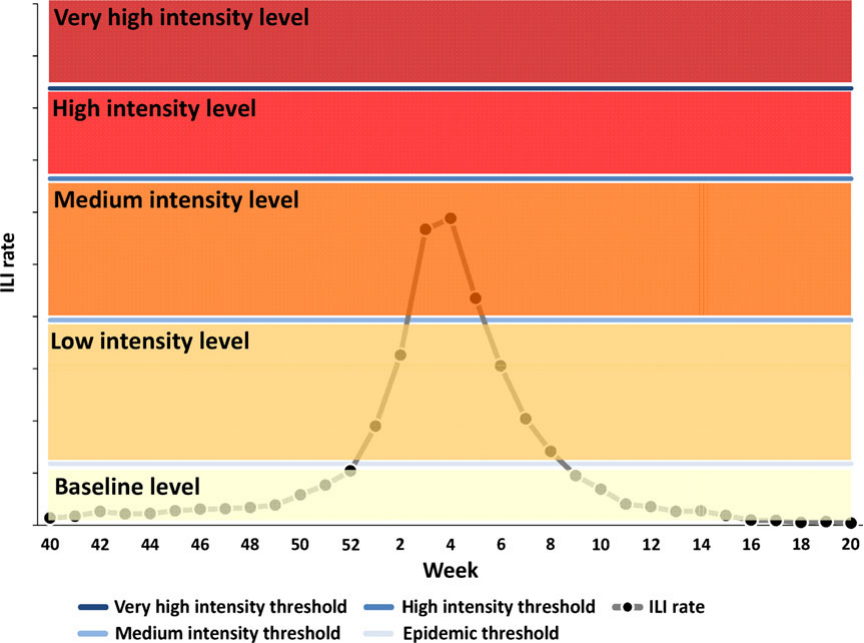
MEM graph model with epidemic and intensity thresholds, intensity levels, and the weekly ILI/ARI rate.– weekly rate > very high intensity threshold.– High intensity threshold < weekly rate ≤ very high intensity threshold.– medium intensity threshold < weekly rate ≤ high intensity threshold.– epidemic threshold < weekly rate ≤ medium intensity threshold.– weekly rate ≤ epidemic threshold. ILI, influenza-like illnesses; ARI, acute respiratory illnesses; MEM, moving epidemic method.

The intensity levels were defined as follows (Figure1):

1. Baseline: weekly rate ≤ epidemic threshold.
2. Low: epidemic threshold < weekly rate ≤ medium intensity threshold.
3. Medium: medium intensity threshold < weekly rate ≤ high intensity threshold.
4. High: high intensity threshold < weekly rate ≤ very high intensity threshold.
5. Very high: weekly rate > very high intensity threshold.

For the analysis, at least 5 years of consecutive data were required to calculate the threshold and intensity levels for the next season; that is, the analysis started with data from seasons 1996/1997 to 2000/2001 to estimate the thresholds for season 2001/2002, or from the first season available in the country to the fifth one, to estimate the thresholds for the sixth season. From then on, calculations for each subsequent season included one more season of data (to a maximum of 10) to estimate the thresholds. The last step of analysis used data from a maximum of 10 seasons if available (2002/2003 to 2012/2013, excluding the 2009 A(H1N1) pandemic) or at least five seasons (2007/2008 to 2012/2013, excluding the pandemic) to make estimations for season 2013/2014. As we excluded the pandemic season from MEM calculations, estimations for the 2009/2010 and 2010/2011 season thresholds are the same.

For each country, the highest weekly rate per season (the season peak) was compared to the intensity thresholds and described for each of the countries over time. Furthermore, a log scale of the weekly incidence rates and percentage of consultations was used to graphically compare and discuss the season 2013/2014 country intensity levels in Europe.

Finally, weekly maps were drawn to show the spread of the 2013/2014 season intensity in Europe.

Complementary figures in Appendix S1 show the historical data included in this study, the threshold trend over the years, and the season 2013/2014 surveillance. Appendix S2 is an animated gif of the evolution of the 2013/2014 intensity levels by country.

The R Language (v3.2.0) mem library (v1.4) was used for calculations of the thresholds and graphic output.

## Results

### Data

A total of 32 countries fulfilled the inclusion criteria and were asked to participate in the project. One country did not participate and three countries did not reply; 28 countries agreed to participate in this project and authorized the use of their data.

Influenza-like illnesses or ARI weekly data were available in 30 datasets (Table1). Luxembourg and Greece provided percentage of ILI consultations and Switzerland provided both ILI incidence and percentage of ILI consultations. Romania had two datasets, one for ILI and another for ARI. In eight countries, the dataset contained 18 seasons (from 1996/1997 to 2013/2014). The minimum number of seasons available for analysis was eight, in Serbia and Slovakia.

**Table 1:** Countries, type of reported data, and seasons included in the study

Country	Data type	No. of seasons*	Seasons
Belgium	ILI weekly rates	14	2000/2001–2013/2014
Denmark	ILI weekly rates	18	1996/1997–2013/2014
Estonia	ILI weekly rates	9	2005/2006–2013/2014
Greece	ILI % of consultations	10	2004/2005–2013/2014
Hungary	ILI weekly rates	11	2003/2004–2013/2014
Ireland	ILI weekly rates	14	2000/2001–2013/2014
Israel	ILI weekly rates	11	2003/2004–2013/2014
Lithuania	ILI weekly rates	9	2005/2006–2013/2014
Luxembourg	ILI % of consultations	11	2003/2004–2013/2014
Norway	ILI weekly rates	9	2005/2006–2013/2014
Poland	ILI weekly rates	13	2001/2002–2013/2014
Portugal	ILI weekly rates	17	1997/1998–2013/2014
Romania	ILI weekly rates	10	2004/2005–2013/2014
Serbia	ILI weekly rates	8	2006/2007–2013/2014
Slovakia	ILI weekly rates	8	2006/2007–2013/2014
Spain	ILI weekly rates	18	1996/1997–2013/2014
Sweden	ILI weekly rates	10	2000/2001–2002/2003, 2006/2007–2013/2014
Switzerland (con)	ILI % of consultations	18	1996/1997–2013/2014
Switzerland (pop)	ILI weekly rates	14	2000/2001–2013/2014
The Netherlands	ILI weekly rates	18	1996/1997–2013/2014
United Kingdom (ENG)	ILI weekly rates	18	1996/1997–2013/2014
United Kingdom (NIR)	ILI weekly rates	13	2001/2002–2013/2014
United Kingdom (SCT)	ILI weekly rates	11	1996/1997–2013/2014
Albania	ARI weekly rates	15	1999/2000–2013/2014
France	ARI weekly rates	18	1996/1997–2013/2014
Kazakhstan	ARI weekly rates	12	2002/2003–2013/2014
Kyrgyzstan	ARI weekly rates	9	2005/2006–2013/2014
Romania	ARI weekly rates	9	2005/2006–2013/2014
Russian Federation	ARI weekly rates	18	1996/1997–2013/2014
Ukraine	ARI weekly rates	18	1996/1997–2013/2014

ILI, influenza-like illness; ARI, acute respiratory infection.

* Number of seasons available for the study.

### Epidemic and intensity thresholds

Influenza-like illnesses and ARI weekly incidence rates varied considerably by country and season and resulted in large differences in the calculated thresholds (Table2a). The lowest ILI epidemic thresholds (in consultations per 100 000 persons) were observed in Sweden (range, 6·2–8·7), Romania (5·3–13·9), and Estonia (7·4–9·6). Conversely, countries such as Belgium, Denmark, Hungary, Poland, Serbia, and Slovakia had epidemic thresholds above 100 in all the seasons for which data were available.

**Table 2:** (a) Epidemic and intensity thresholds by season and country reporting influenza-like illness (ILI) consultation rate, 2001/2002 to 2013/2014. (b) Epidemic and intensity thresholds by season and country reporting acute respiratory infection (ARI) consultation rate, 2001/2002 to 2013/2014

Country	2001/2002	2002/2003	2003/2004	2004/2005	2005/2006	2006/2007	2007/2008	2008/2009	2009/2010	2010/2011	2011/2012	2012/2013	2013/2014
Epidemic threshold															
Medium intensity threshold															
High intensity threshold															
Very high intensity threshold															
*(a)*															
Belgium	–	–	–	–	167·0	168·2	172·1	170·5	176·5	176·5	173·0	168·9	178·5
	–	–	–	–	371·7	390·2	430·6	413·6	463·5	463·5	462·1	495·1	499·8
	–	–	–	–	702·5	700·7	770·9	732·9	861·1	861·1	831·8	821·5	855·4
					930·8	907·6	997·3	943·8	1132·2	1132·2	1078·7	1027·5	1084·7
Denmark	179·6	177·4	176·3	171·7	170·7	166·6	160·0	160·7	135·6	135·6	135·5	115·2	114·8
	290·6	274·7	294·1	295·1	298·9	290·1	277·4	250·1	235·6	235·6	235·4	191·1	194·6
	482·0	489·4	518·6	506·5	521·7	502·1	473·3	497·2	413·4	413·4	412·8	415·1	420·7
	602·7	631·7	666·3	643·2	667·3	639·9	599·3	673·6	530·1	530·1	529·2	584·9	591·6
Estonia	–	–	–	–	–	–	–	–	–	–	7·4	8·2	9·6
	–	–	–	–	–	–	–	–	–	–	14·6	15·0	17·0
	–	–	–	–	–	–	–	–	–	–	53·5	48·8	51·2
	–	–	–	–	–	–	–	–	–	–	95·0	82·1	83·3
Greece*	–	–	–	–	–	–	–	–	1·4	1·4	1·6	1·8	1·8
	–	–	–	–	–	–	–	–	3·5	3·5	3·9	4·4	4·2
	–	–	–	–	–	–	–	–	5·0	5·0	5·9	6·6	6·4
									5·9	5·9	7·1	8·0	7·8
Hungary	–	–	–	–	–	–	–	146·0	149·9	149·9	149·8	146·3	145·4
	–	–	–	–	–	–	–	283·4	327·6	327·6	373·0	365·4	389·0
	–	–	–	–	–	–	–	595·6	620·9	620·9	672·5	644·8	660·8
	–	–	–	–	–	–	–	826·9	823·6	823·6	872·7	828·7	835·2
Ireland	–	–	–	–	26·0	25·5	25·5	24·7	24·6	24·6	24·6	19·2	18·5
	–	–	–	–	40·0	43·8	47·9	46·7	53·9	53·9	58·3	52·1	57·1
	–	–	–	–	94·3	96·0	98·4	93·1	107·1	107·1	130·2	115·3	114·2
	–	–	–	–	137·7	135·8	135·3	126·4	145·1	145·1	185·6	163·8	155·2
Israel	–	–	–	–	–	–	–	28·9	28·8	28·8	34·3	33·4	36·2
	–	–	–	–	–	–	–	107·1	110·2	110·2	129·0	117·0	128·7
	–	–	–	–	–	–	–	241·3	236·6	236·6	275·6	260·8	281·1
	–	–	–	–	–	–	–	345·4	331·7	331·7	385·5	371·7	397·0
Lithuania	–	–	–	–	–	–	–	–	–	–	11·2	11·2	14·2
	–	–	–	–	–	–	–	–	–	–	96·8	75·1	99·9
	–	–	–	–	–	–	–	–	–	–	291·0	354·7	443·7
	–	–	–	–	–	–	–	–	–	–	473·3	704·3	857·9
Luxembourg*	–	–	–	–	–	–	–	1·4	1·5	1·5	1·7	1·7	1·8
	–	–	–	–	–	–	–	2·8	3·3	3·3	4·2	4·1	4·8
	–	–	–	–	–	–	–	6·4	7·3	7·3	9·0	8·6	9·5
	–	–	–	–	–	–	–	9·3	10·3	10·3	12·7	11·9	12·9
Norway	–	–	–	–	–	–	–	–	–	–	52·3	54·0	66·0
	–	–	–	–	–	–	–	–	–	–	140·6	146·7	160·2
	–	–	–	–	–	–	–	–	–	–	239·2	238·4	266·9
	–	–	–	–	–	–	–	–	–	–	302·5	295·5	334·5
Poland	–	–	–	–	–	109·6	111·9	117·2	112·7	112·7	112·8	121·1	139·2
	–	–	–	–	–	126·7	146·3	156·4	167·3	167·3	180·4	178·8	229·4
	–	–	–	–	–	576·0	597·5	583·9	594·3	594·3	584·2	549·1	598·1
	–	–	–	–	–	1124·8	1112·8	1045·3	1040·8	1040·8	981·9	901·4	913·6
Portugal	–	19·1	19·7	20·6	21·6	22·6	22·3	22·9	31·2	31·2	30·3	31·6	32·8
	–	28·0	29·5	35·6	40·0	39·7	42·5	48·1	48·6	48·6	60·0	70·0	66·2
	–	94·7	93·6	120·5	139·5	136·0	141·5	144·2	149·9	149·9	147·1	161·6	151·4
	–	162·2	156·0	206·5	242·4	234·3	240·8	234·4	246·6	246·6	218·8	233·9	218·2
Romania	–	–	–	–	–	–	–	–	5·3	5·3	14·1	13·9	13·1
	–	–	–	–	–	–	–	–	6·9	6·9	14·1	13·9	13·1
	–	–	–	–	–	–	–	–	21·0	21·0	37·1	34·6	31·1
	–	–	–	–	–	–	–	–	34·4	34·4	68·7	62·7	54·5
Serbia	–	–	–	–	–	–	–	–	–	–	–	103·6	100·5
	–	–	–	–	–	–	–	–	–	–	–	149·1	163·8
	–	–	–	–	–	–	–	–	–	–	–	291·8	302·6
	–	–	–	–	–	–	–	–	–	–	–	392·6	396·8
Slovakia	–	–	–	–	–	–	–	–	–	–	–	264·4	265·1
	–	–	–	–	–	–	–	–	–	–	–	398·8	434·3
	–	–	–	–	–	–	–	–	–	–	–	792·8	832·3
	–	–	–	–	–	–	–	–	–	–	–	1074·0	1109·6
Spain	81·8	81·0	80·7	78·7	80·6	78·0	76·0	69·6	54·7	54·7	54·9	58·4	58·9
	151·5	173·2	166·6	168·6	191·8	186·1	184·6	178·7	168·9	168·9	160·2	204·4	196·6
	575·7	609·4	566·3	528·7	609·3	564·4	558·6	539·5	487·7	487·7	431·0	365·6	332·2
	1038·7	1062·7	972·6	876·3	1015·7	921·5	911·3	879·2	779·3	779·3	667·5	472·9	418·8
Sweden	–	–	–	–	–	–	6·2	6·4	6·5	6·5	6·7	8·7	8·6
	–	–	–	–	–	–	16·2	16·5	17·5	17·5	17·7	19·8	20·0
	–	–	–	–	–	–	41·0	38·3	37·6	37·6	36·3	39·5	38·6
	–	–	–	–	–	–	61·9	55·5	52·8	52·8	49·8	53·6	51·5
Switzerland (con)*	1·2	1·2	1·2	1·2	1·2	1·2	1·1	1·0	0·9	0·9	0·9	0·9	0·9
	4·0	4·2	4·4	4·3	4·6	4·1	4·0	3·7	3·6	3·6	3·4	3·4	3·4
	8·0	7·6	7·3	7·2	7·4	7·8	7·5	7·1	6·5	6·5	5·6	5·7	5·9
	10·7	10·0	9·2	9·1	9·2	10·3	10·0	9·4	8·4	8·4	7·0	7·1	7·5
Switzerland (pop)	–	–	–	–	65·4	64·9	66·8	65·1	67·1	67·1	68·1	66·5	72·9
	–	–	–	–	258·9	241·3	265·1	265·0	294·5	294·5	292·9	279·9	282·8
	–	–	–	–	423·0	438·1	452·9	438·4	486·4	486·4	472·2	478·3	492·3
	–	–	–	–	525·5	570·3	573·9	547·6	607·1	607·1	583·2	606·2	629·0
The Netherlands	54·7	55·6	56·1	57·8	60·3	58·9	56·9	56·8	50·8	50·8	49·0	49·2	52·4
	118·7	122·3	111·7	113·9	125·9	125·4	110·1	101·6	99·0	99·0	91·1	91·1	92·2
	279·5	269·2	276·5	267·6	292·3	279·3	247·7	238·5	224·9	224·9	175·4	175·9	180·6
	408·1	381·4	412·7	390·4	424·0	397·7	354·6	347·7	323·2	323·2	234·4	235·3	243·0
United Kingdom (ENG)	42·1	41·7	41·6	40·6	40·4	39·2	37·5	28·5	25·5	25·5	19·3	16·1	15·3
	86·3	77·5	66·9	64·5	62·2	58·8	51·2	46·5	42·5	42·5	41·7	35·6	34·2
	192·9	196·1	203·8	185·7	190·9	176·4	134·1	130·0	98·4	98·4	78·2	75·1	72·9
	275·2	295·5	333·4	296·2	313·4	286·7	205·2	204·9	142·6	142·6	103·3	104·4	101·9
United Kingdom (NIR)	–	–	–	–	–	41·6	54·7	53·8	52·6	52·6	52·5	52·4	52·1
	–	–	–	–	–	61·1	72·2	73·7	79·8	79·8	90·0	79·3	85·7
	–	–	–	–	–	122·6	166·6	163·3	182·7	182·7	219·0	213·0	209·7
	–	–	–	–	–	166·8	241·0	232·2	263·3	263·3	324·3	329·6	311·4
United Kingdom (SCT)	–	–	–	–	–	–	–	39·0	42·4	42·4	43·0	41·3	43·2
	–	–	–	–	–	–	–	92·5	105·8	105·8	120·4	83·6	84·2
	–	–	–	–	–	–	–	171·8	211·3	211·3	235·0	296·3	307·4
	–	–	–	–	–	–	–	225·7	286·9	286·9	315·9	518·3	544·8
*(b)*													
Albania	–	–	–	522·9	530·1	526·2	526·8	521·7	512·0	512·0	472·7	451·3	413·8
	–	–	–	569·4	568·0	553·4	547·2	554·0	544·2	544·2	529·1	527·1	519·2
	–	–	–	673·1	670·4	680·7	669·1	669·6	662·0	662·0	632·8	626·8	613·0
	–	–	–	724·7	721·3	746·0	731·3	728·1	721·8	721·8	684·9	676·6	659·7
France	2255·4	2250·3	2274·4	2274·4	2265·3	2240·2	2277·9	2277·9	2225·3	2225·3	2197·4	2197·4	2143·0
	3165·9	3285·2	3332·1	3393·4	3512·2	3458·1	3359·9	3266·6	3187·8	3187·8	3116·4	3087·0	3050·7
	4077·0	4179·0	4199·0	4268·0	4420·2	4351·5	4187·8	4208·5	4033·5	4033·5	3958·2	3983·2	3872·9
	4559·1	4647·9	4650·9	4723·2	4893·1	4816·7	4616·0	4707·2	4475·5	4475·5	4399·4	4458·2	4303·7
Kazakhstan	–	–	–	–	–	–	231·9	231·7	233·8	233·8	231·2	234·6	233·5
	–	–	–	–	–	–	376·9	378·4	384·7	384·7	379·3	377·8	357·3
	–	–	–	–	–	–	730·4	721·9	727·3	727·3	693·7	713·7	685·7
	–	–	–	–	–	–	978·6	960·4	963·8	963·8	905·8	945·5	914·7
Kyrgyzstan	–	–	–	–	–	–	–	–	–	–	77·4	75·2	73·9
	–	–	–	–	–	–	–	–	–	–	106·7	115·0	114·0
	–	–	–	–	–	–	–	–	–	–	191·6	205·0	210·1
	–	–	–	–	–	–	–	–	–	–	248·1	264·6	275·3
Romania	–	–	–	–	–	–	–	–	–	–	697·4	794·3	796·9
	–	–	–	–	–	–	–	–	–	–	772·3	807·6	844·5
	–	–	–	–	–	–	–	–	–	–	932·2	949·2	977·8
	–	–	–	–	–	–	–	–	–	–	1013·0	1019·5	1043·2
Russian Federation	716·1	716·9	716·7	715·8	720·7	718·0	716·2	713·5	695·7	695·7	700·4	675·5	691·2
	1016·2	1017·1	1071·0	1045·0	1043·6	1009·9	972·1	948·1	911·3	911·3	912·6	888·8	902·7
	1429·0	1427·9	1467·3	1428·5	1452·8	1425·6	1342·5	1328·9	1162·1	1162·1	1178·1	1172·2	1196·0
	1661·4	1658·9	1686·3	1640·2	1681·6	1660·2	1548·3	1542·7	1293·9	1293·9	1318·9	1324·8	1354·4
Ukraine	675·6	682·8	682·8	703·4	702·7	693·6	691·3	685·9	643·0	643·0	623·2	591·0	588·0
	946·6	943·1	1013·2	961·1	999·8	939·6	934·1	918·8	847·8	847·8	808·9	763·6	755·6
	1469·2	1469·9	1567·7	1516·6	1572·9	1548·4	1531·3	1528·9	1382·6	1382·6	1218·3	1110·8	1109·0
	1784·3	1788·4	1901·3	1855·5	1921·6	1930·9	1905·2	1914·7	1716·2	1716·2	1460·0	1310·9	1314·0

* Percentage of consultation due to ILI.

Differences among medium to high and very high intensity thresholds are consistently uniform across time in most countries. Belgium had the highest very high intensity threshold in 2009/2010 and 2010/2011, followed by Poland in 2006/2007, Slovakia in 2013/2014, and Spain in 2002/2003.

Countries reporting percentage consultations due to ILI, such as Greece, Luxembourg, and Switzerland, presented similar thresholds, ranging from 0·9% to 1·8% for the epidemic threshold through the seasons, to more than 10% for the very high intensity threshold in some seasons in Luxemburg and Switzerland.

ARI incidence rate thresholds differed as much across countries as ILI thresholds did (Table2b). Kyrgyzstan had the lowest epidemic threshold and France the highest. Very high intensity ARI thresholds ranged from 248 (Kyrgyzstan 2011/2012) to 4893 (France 2005/2006).

Moving epidemic method ILI thresholds showed a wider range than ARI thresholds with very high intensity thresholds exceeding epidemic thresholds 4- to 11-fold and 1- to 3·5-fold for ILI and ARI, respectively, depending on the country and season. The variations in the MEM thresholds over time are shown by country in Appendix S1.

### Trend in intensity levels across seasons by country

In Norway and the United Kingdom (England and Northern Ireland), two differentiated waves were observed in season 2009/2010. In these cases, the highest weekly rate in the full season (including both waves) was considered as the ‘peak rate’.

The countries reporting ILI (Table3a) with seasons at the top intensity levels were Greece and the United Kingdom (Northern Ireland), three seasons each; Ireland, two seasons; and Luxembourg, Norway, Romania, and the United Kingdom (England and Scotland), one each. In contrast, some countries did not reach the epidemic thresholds at any time during some seasons, particularly during the last season 2013/2014, when peaks remained at the baseline level in Denmark, Romania, Serbia, Slovakia, and the United Kingdom (England, Northern Ireland, and Scotland).

**Table 3:**
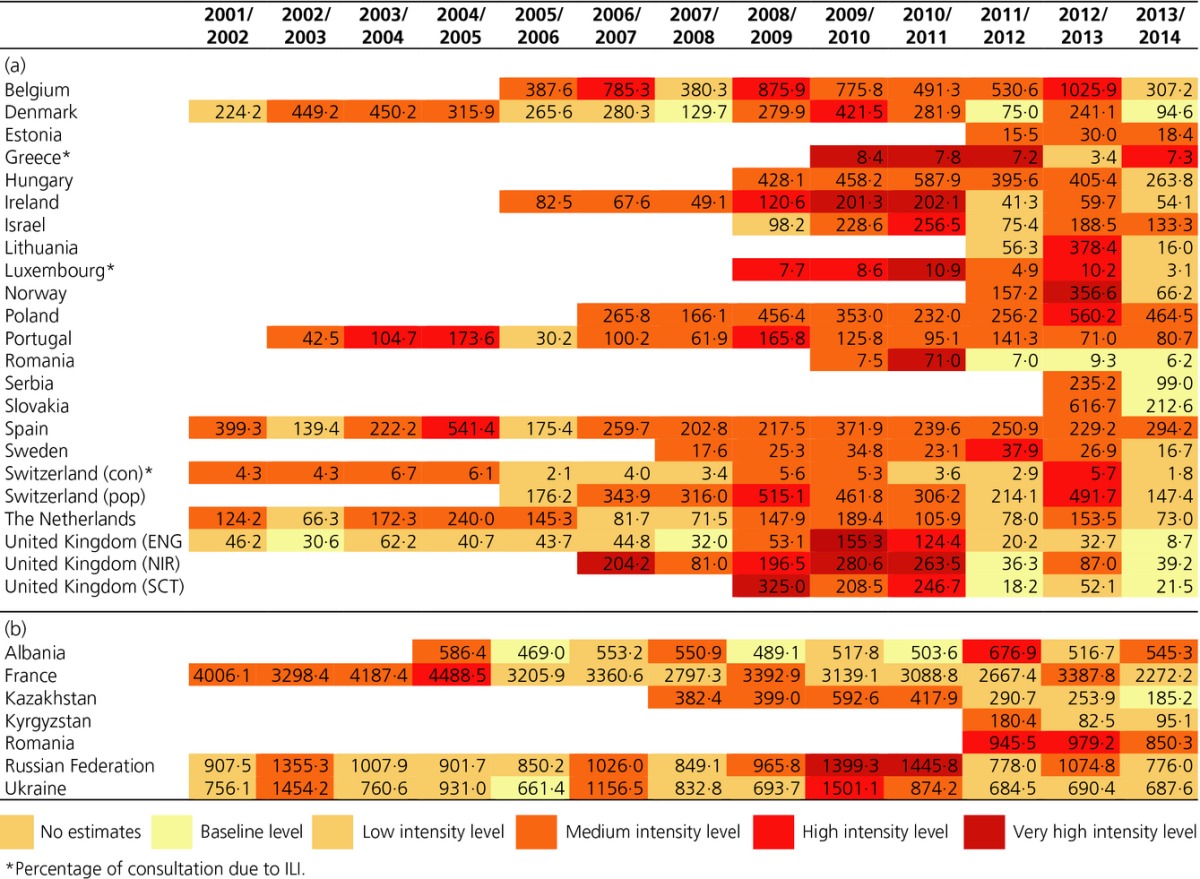
(a) Highest influenza-like illness (ILI) weekly consultation rate intensity by season and country (colors indicate the ILI intensity level corresponding to the highest rate in the season), 2001/2002 to 2013/2014. (b) Highest acute respiratory infections (ARI) weekly consultation rate intensity by season and country (colors indicate the ARI intensity level corresponding to the highest rate in the season), 2001/2002 to 2013/2014

Season 2009/2010 was the most intense according to MEM peak intensity. In the pandemic season, all countries were above the medium intensity threshold, with 35% of them at high or very high intensity levels. High intensity peaks continued through season 2010/2011, with 41% of countries at the two top levels, whereas season 2011/2012 showed a significant decreasing intensity, with 52% of countries at the baseline or low levels and only 5% at high or very high levels.

In countries reporting ARI (Table3b), the intensity levels throughout the countries and seasons were quite similar, with the highest intensity in seasons 2009/2010 and 2010/2011 in the Russian Federation.

### Intensity of the 2013/2014 season

**Figure 2:**
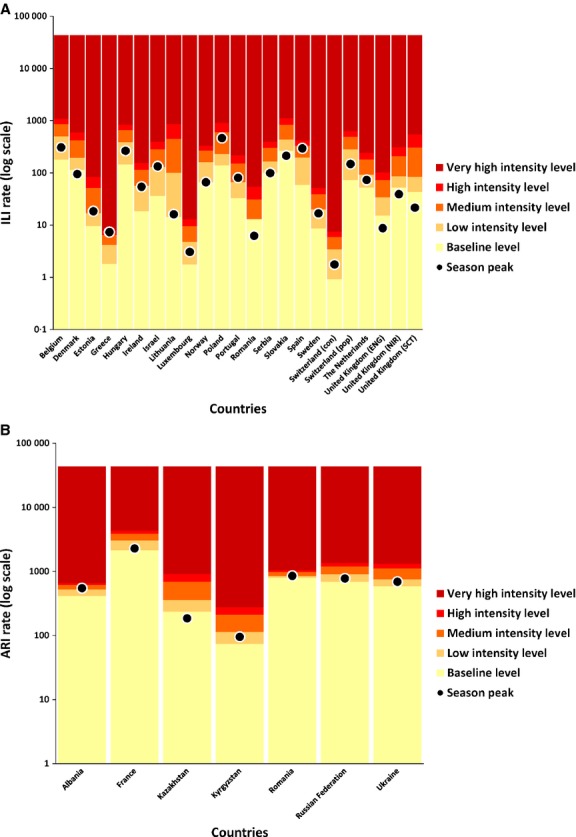
(A) Intensity levels and highest weekly ILI rate by country in the 2013/2014 season. (B) Intensity levels and highest weekly ARI rate by country in the 2013/2014 season. ILI, influenza-like illnesses; ARI, acute respiratory illnesses.

Figure2A,B shows the ILI and ARI intensity levels calculated for season 2013/2014 and the observed peak rate (in a log scale) by country. Poland, Belgium, Spain, and Hungary had the highest weekly ILI consultation rate per 100 000 (464·5, 307·2, 294·2, and 263·8, respectively), and Romania, Lithuania, and Sweden had the lowest (6·2, 16·0, and 16·7, respectively). However, peak weekly consultation rates in Belgium, Hungary, and Sweden, for instance, remained at the same low intensity level. Slovakia, Denmark, Romania, and United Kingdom (England), with peak rates as different as 212·6, 94·6, 6·2, and 8·7, respectively, were at the baseline.

Countries reporting ARI showed larger differences in peak rates compared with those reporting ILI, although the intervals between thresholds were narrower. France (with an ARI peak rate of 2272·2), the Russian Federation (with a peak rate of 776·0), or Kyrgyzstan (with 95·1) were considered to have the same medium intensity level in 2013/2014. During the last three seasons, Romania (which reported both ARI and ILI data) showed baseline to medium intensity levels of ILI consultation rates but medium to high levels of ARI consultation rates.

**Figure 3:**
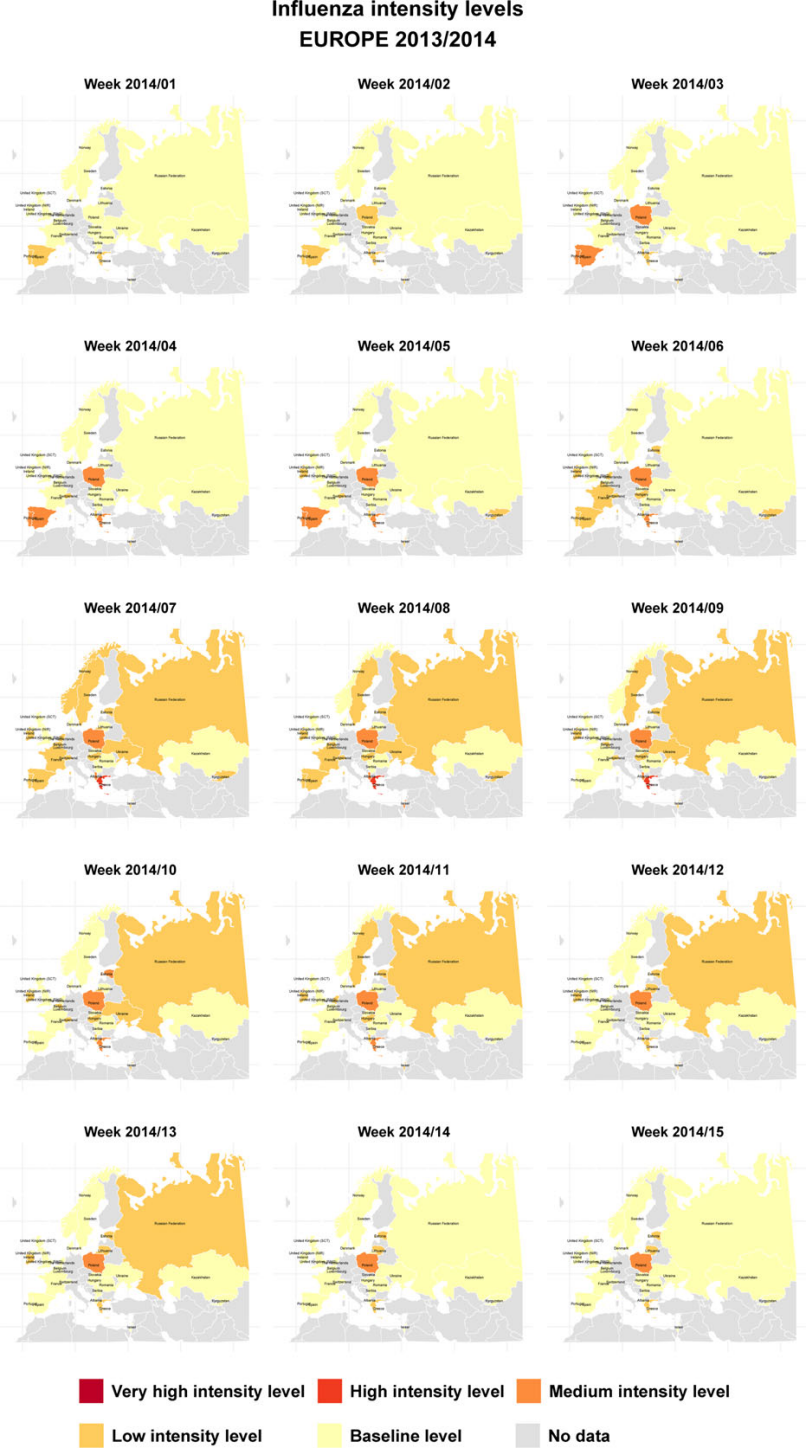
Weekly ILI/ARI intensity levels in Europe 2013/2014 during the epidemic period (weeks 01/2014 to 15/2014). ILI, influenza-like illnesses; ARI, acute respiratory illnesses.

Weekly intensity in Europe during the 2013/2014 epidemic period is represented in Figure3 and in the animated Appendix S2. The epidemic period started in Europe in January 2014 in Poland, Greece, and Spain. Data from Poland show an increase of the weekly rates in the last two seasons with relative high pre-epidemic rates, as happens in countries reporting ARI, which could explain the early start of the epidemic period in this country. The intensity was between low and medium, and only Greece reached high intensity level in weeks 7 to 9/2014.

## Discussion

Estimated rates based on ILI and ARI consultations showed great variability across countries, most likely due to different health systems, healthcare-seeking behavior, and surveillance schemes, including case definitions^15^ and virological surveillance.^16^ The WHO Regional Office for Europe and the ECDC receive weekly information on ILI and/or ARI from surveillance systems using either population or consultation denominators as well as qualitative indicators such as intensity, trend, and geographical spread, by country. Nevertheless, comparing countries is difficult despite efforts to standardize and remains a challenge for global influenza surveillance.

The search for a gold standard epidemic threshold and intensity indicators has been long-standing, particularly as sentinel surveillance systems were put in place in Europe. However, there is no final consensus on the method, in spite of the demands of international organizations and country authorities. Most of the methods implemented to model influenza seasons use *ad hoc* discretionary parameters to establish the epidemic period,^12^,^17^–^20^ which is one of the major constraints to comparability.

In this study, we have used defined criteria to calculate epidemic and intensity thresholds, regardless of the type and quality of the data and their fit to the model. The data suggest that intracountry ARI epidemic rates had fewer variations than ILI rates (Figures S1A), suggesting greater stability of ARI incidence, according to the multiple origins of such infections. Consequently, variance is lower, confidence limits (particularly at 90 and 97·5 CI) are narrower, and ARI intensity thresholds are more stable than ILI (Figures S1B). In contrast, ARI pre-epidemic rates usually vary more, raising epidemic thresholds.

Epidemic and intensity thresholds vary according to the historical rates in each country with important differences, as observed, for instance, in Belgium and Sweden. Although this is an expected finding, these thresholds describe the shape of the epidemic waves and point out the necessity of a standard way to make comparisons. Epidemic thresholds are also influenced by the quality of the surveillance system. Countries with large variations in pre-epidemic ILI rates had high epidemic thresholds which, in the case of Romania, were higher than the medium intensity threshold. Additionally, the extreme variations in ILI peaks increased the upper confidence limits of the intensity thresholds, as observed in Lithuania, the United Kingdom (Scotland), and, again, in Romania and Slovakia.

Although the season peak rate is not the only indicator of seasonal intensity, it is useful for assessing and comparing either intra- or intercountry variations. Other intensity measures (such as the season-cumulative rate or the area under the epidemic period curve) could be useful for comparing seasons and countries, but they are unhelpful for monitoring weekly intensity in real time, which is a surveillance priority.

Moving epidemic method translates quantitative rates (or percentages of consultations) into a standardized qualitative intensity values which permit assessing intercountry differences. ILI peak rates – as different as 307·2 in Belgium, 16·0 in Lithuania, and 16·7 in Sweden in 2013/2014 – were all classified as low intensity. Similarly, Luxemburg, with a peak of 3·1 ILI cases per 100 consultations, and Hungary, with a peak of 263·8 ILI consultations per 100·000 population, would both be classified as *low* intensity in 2013/2014. In contrast, MEM considered the ILI rate of 356·6 in Norway in 2012/2013 to be *very high* and 380·3 in Belgium in 2007/2008 as *low*.

Influenza-like illnesses intensity thresholds tended to decline as a consequence of the general trend of influenza in the last decade in Europe. However, an intense season could increase variability, raise intensity thresholds, and lead to similar rates qualifying as medium in one season and high in the following one, as occurred in Albania with 553·2 and 550·9 in 2006/2007 and 2007/2008, respectively, reflecting the general trend and locating the current season intensity in the epidemic context of the past years. From the geographical point of view, rates in some large countries could vary considerably from one region to another.^21^ In these countries, such as the Russian Federation, for instance, the thresholds should be calculated at the regional level to give more accurate information.

The number of seasons included in the calculation, however, remains a point of discussion. The minimum number for obtaining stable thresholds was established at five, and the maximum was limited to 10, to remain in the latest trend, quite changing in some European countries.^22^,^23^ The pandemic season 2009/2010 was omitted because of its special surveillance characteristics with aberrant rate estimates in some countries. MEM thresholds must be updated on a yearly basis which, more than an annoyance, is an opportunity to include the latest trend.

The cut points are just a reference for comparison and do not affect the meaning of the results. The confidence intervals (40%, 90% and 97·5%) used by MEM to establish the intensity limits have been selected after a descriptive analysis of all seasons and countries and a final agreement on the better distribution of the intensity levels in Europe.^24^

Some of the principal limitations of this method are associated with changes of the surveillance system or in access to primary are, but these problems are common to all modeling approaches. MEM detects erroneous or outlier data in the series (as in the 2009/2010 pandemic season), allowing to decide whether or not to include them in the calculation.

Other quantitative indicators could also assess the intensity. The cumulative ILI or ARI incidence rate in a season would be one possibility. However, this rate can only be estimated at the end of the season, which limits its use in monitoring. Moreover, as the duration of the epidemic periods is quite similar, particularly in the case of ILI, weekly intensity rates (including the peak rate) are a good proxy for the overall intensity of one season.

New approaches for intensity assessment should include virological information to confirm and quantify virus circulation and to predict epidemic size.^25^ The percentage of influenza virus detection could be included for assessing ILI epidemic intensity, as some authors^26^ have already done, as well as standard registers for studying the severity of cases. Nevertheless, assuming that these figures could reliably reflect the epidemic activity in a country, these indicators are conditioned by the health system, health resources, laboratory capacity, and health practice, which makes comparison almost impossible. In the 2013–2014 season, principal influenza strains circulating in Europe were A/California/7/2009 (H1N1)-like (58% of all isolations), A/Texas/50/2012 (H3N2)-like (38%), and type B/Yamagata lineage viruses (4%), and no major differences were observed between countries that could explain the weekly rate variations.

Although MEM was primarily developed for modeling ILI data, it has been used successfully with ARI data.^14^ MEM thresholds suggest that ARI waves are quite different from ILI ones, with higher epidemic thresholds and shorter range among intensity thresholds. ILI, mainly caused by the influenza virus, has much higher epidemic waves than ARI and lower and stable pre-epidemic weekly rates. A large distance from epidemic threshold to very high intensity threshold in ILI is consistent with this (high range between thresholds). On the contrary, ARI is caused by a wide number of different microorganisms which leads to a great variation in pre-epidemic rates and to low epidemic waves with similar shapes each year (low range between thresholds). In these cases, complementary virological information could be necessary for determining the start of the epidemic wave to avoid false alarms. Laboratory-confirmed respiratory infection outbreaks or any important increase in positive swabs should be monitored to evaluate the overall influenza epidemic situation.

Intensity, understood as the level of the population consultation rates or the percentage of ILI or ARI primary care consultations, is not synonymous with activity or severity, but is one of the most valid and reliable indicators for the impact of influenza on the population. Comparing intensity across seasons or countries is essential for understanding the epidemic patterns of seasonal epidemics and future pandemics, and for evaluating control measures, such as the effectiveness of vaccination campaigns. Consequently, an automated standardized model for comparison should replace the subjective intensity reports in surveillance systems at national and international levels.

## Supporting Information

**Appendix S1**. Historical data included in the study, thresholds trend over the years and season 2013/2014 by country.

**Appendix S2**. Animated gif of the evolution of the Influenza-like illness or acute respiratory infection intensity in Europe during the 2013/2014 season.
