# Supplementary figures and images for: Influenza surveillance in Europe: comparing intensity levels calculated using the moving epidemic method
Source: Influenza Other Respir Viruses. 2015 Aug 4;9(5):234–46. doi: 10.1111/irv.12330 (PMC4548993; doi:10.1111/irv.12330)

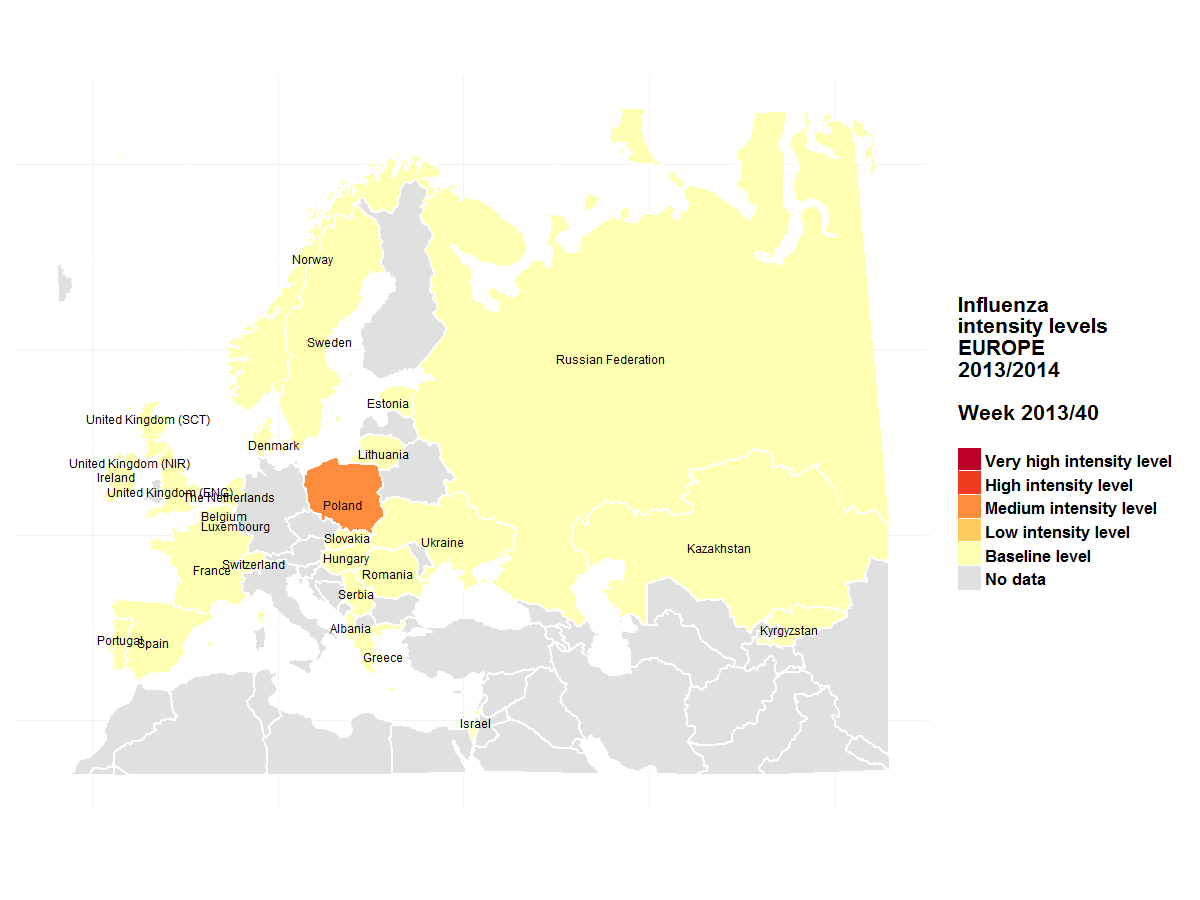

Supplement: Supplementary file 2 [file irv0009-0234-sd2.gif]
